# High Levels of Kinesiophobia at Discharge from the Hospital May Negatively Affect the Short-Term Functional Outcome of Patients Who Have Undergone Knee Replacement Surgery

**DOI:** 10.3390/jcm9030738

**Published:** 2020-03-09

**Authors:** Henri De Vroey, Kurt Claeys, Keivan Shariatmadar, Ive Weygers, Evie Vereecke, Geert Van Damme, Hans Hallez, Filip Staes

**Affiliations:** 1 KU Leuven, Campus Bruges, Department of Rehabilitation Sciences, Spoorwegstraat 12, 8200 Bruges, Belgium; Kurt.claeys@kuleuven.be (K.C.); ive.weygers@kuleuven.be (I.W.); 2 KU Leuven, Campus Bruges, Department of Mechanical Engineering, Spoorwegstraat 12, 8200 Bruges, Belgium; keivan.shariatmadar@kuleuven.be; 3 KU Leuven, Campus Kulak Kortrijk, Department of Development and Regeneration, Etienne Sabbelaan 53, 8500 Kortrijk, Belgium; evie.vereecke@kuleuven.be; 4 AZ Sint Lucas Hospital, Department of Orthopedic Surgery, Sint-Lucaslaan 29, 8310 Bruges, Belgium; geert.vandamme@stlucas.be; 5 KU Leuven, Campus Bruges, Department of Computer Science, Spoorwegstraat 12, 8200 Bruges, Belgium; hans.hallez@kuleuven.be; 6 KU Leuven, Campus Leuven, Department of Rehabilitation Sciences, Tervuursevest 101, 3001 Leuven, Belgium; filip.staes@kuleuven.be

**Keywords:** patient-reported outcome, performance-based measure, movement-related fear, kinesiophobia, knee arthroplasty, rehabilitation

## Abstract

Background: Kinesiophobia is a psycho-cognitive factor that hampers recovery after orthopedic surgery. No evidence exists on the influence of kinesiophobia on the short-term recovery of function in patients with knee replacement (KR). Therefore, the aim of the present study is to investigate the impact of kinesiophobia on short-term patient-reported outcomes (PROMs) and performance-based measures (PBMs). Methods: Forty-three KR patients filled in the Tampa scale for kinesiophobia (TSK) at time of discharge. Patients with TSK ≥ 37 were allocated to the kinesiophobia group (*n* = 24), others to the no-kinesiophobia group (*n* = 19). Patients were asked to complete PROMs and to execute PBMs at discharge and at 6-weeks follow-up. An independent samples t-test was used to compare group differences for PROMs and PBMs at both measurement sessions. Multiple linear regression analysis models were used to model PBM outcomes from age, pain and TSK scores. Results: Significant differences were observed between groups for PROMs and PBMs. Kinesiophobia significantly contributed to the reduced functional outcomes. Conclusion: At discharge from the hospital, 55.8% of KR patients demonstrated high levels of kinesiophobia (TSK ≥ 37). This may negatively influence short-term recovery of these patients, by putting them at higher risk for falling and reduced functionality.

## 1. Introduction

Knee replacement (KR) is an effective treatment option for symptoms of end-stage knee osteoarthritis [1]. Despite the high success rate of this surgical procedure, up to 20% of patients report dissatisfaction with the results of their surgery [2]. Moreover, due to persistent pain and disability, a more sedentary life style has been observed in knee replacement patients, putting them at higher risk for both mental and chronic physical illness compared to the general population [3]. Although potential mechanisms that underlie patient dissatisfaction after knee replacement surgery have been suggested by previous authors [4,5], no clear consensus could be found in the literature regarding this issue.

Kinesiophobia or fear of movement (FOM) is a well-known psychological factor that can influence patient outcome and recovery after trauma or surgery at the knee [6,7,8]. Patients with FOM are more likely to be physically inactive and depressed [9]. This results in a disuse syndrome, which can delay normal tissue healing and impede further recovery [10,11]. Kinesiophobia also has a crucial role in the development of hypervigilance, a state in which a patient will perceive normal sensory sensations (e.g., touch) as threatening and harmful. This mental state mediates the chronification of pain through neuroplastic changes in brain regions responsible for behavior and the processing of sensory information [12,13]. Considering the evidence mentioned above, it seems imperative that kinesiophobia is assessed in orthopedic patients, as it will enable clinicians to provide a personalized treatment approach to patients that suffer from movement-related fear [14].

The Tampa scale for Kinesiophobia (TSK) is a validated questionnaire that is widely used for the evaluation of fear and, more specifically, pain-related Kinesiophobia in subjects with a musculoskeletal disorder (e.g., chronic pain, osteoarthritis, low back pain, neck pain) [15,16,17,18]. As much as 25% of all knee arthroplasty patients show elevated levels of FOM [19]. Female sex, lower levels of education, negative coping styles, and older age were all associated with the development of postoperative kinesiophobia [20]. For instance, kinesiophobia has already been associated with a reduced knee flexion range of motion among knee replacement patients [8,14]. Previous research has also highlighted that 60% of the increase in pain score could be related to the presence of kinesiophobia [21,22]. This was supported by findings from a large cohort study demonstrating that, independent from other psychological factors, kinesiophobia negatively influences the mental state, function and the ability to participate in social activities in patients with TKA [21]. This implies that kinesiophobia affects the biopsychosocial well-being of TKA patients. However, results reported above are from cross-sectional design studies. There are no studies that focus on the impact of kinesiophobia on the functional recovery of knee replacement patients using a longitudinal follow-up design. Furthermore, most of these studies rely solely on patient-reported outcome measures (PROMs), which are questionnaire-based evaluations of self-perceived function. These questionnaires are often biased by psycho-cognitive factors, such as depression or anxiety, and might therefore not be representative for the actual functional capabilities of a patient [23,24]. In contrast, the use of performance-based measures (PBMs), of which the Timed Up and Go (TUG) test and Tinetti test are frequently used within different populations, might be more appropriate to capture the actual functionality of patients recovering from knee replacement surgery [24,25]. The Tinetti test comprises a gait and balance score, which represent a patient’s overall risk of falling [26]. If the Tinetti outcome is lower than 19, a patient will have a fivefold risk of falling. During a TUG test, the time a patient requires to perform several dynamic transitions is evaluated. Similarly to the Tinetti test, the TUG is also based on the principle of benchmarking, to which lower extremity function and fall risk are related. More time to complete this test is related to a worse functionality [27,28,29].

The general aim of the present study is to assess the impact of kinesiophobia on the short-term recovery of function after knee replacement at discharge from the hospital and at 6-weeks follow-up. We hypothesize that a relation exists between the presence of kinesiophobia and the short-term outcomes of patients who have undergone knee replacement surgery. We also expect that, similar to other populations with musculoskeletal disorders, kinesiophobia will negatively influence the outcomes of PBMs.

## 2. Experimental Section

### 2.1. Subjects

Forty-three subjects with a knee replacement (age: 49–83) were recruited over a 2-year period. All subjects were operated on by the same surgeon (GVD) in the same hospital. Patients with a history of trauma to the lower limbs or neurologic or systemic disease that might affect gait and balance were excluded from this study. All subjects provided a written informed consent (ethical commission KUL). All test procedures were performed according to the declaration of Helsinki. Table 1 provides the patients’ characteristics.

### 2.2. Experimental Procedure

#### 2.2.1. Data Collection

Subjects were asked to fill in PROMs and execute PBMs at discharge (3 days post-surgery) at 6 weeks post knee replacement surgery. Data were collected by the same experienced physiotherapist (HDV) in a dedicated room at the hospital at time of discharge or at the gait analysis laboratory located at the university campus at follow-up. In addition, a comparison was conducted in which the PROMs and PBMs were compared between patients with TKA and UKA. No significant difference could be demonstrated regarding these outcome measures based on arthroplasty design. 

#### 2.2.2. Patient-Reported Outcome Measures

The Tampa Scale for Kinesiophobia (TSK), Knee Osteoarthritis Outcome Score (KOOS), the Oxford Knee Score (OKS), and visual analogue scale (VAS) evaluating pain were used as PROMs. The TSK is a 17-item questionnaire that is widely used for the assessment of kinesiophobia and fear of (re)injury across various types of musculoskeletal conditions [7,30,31]. A previously validated cut-off score of 37 on the TSK was set in order to allocate knee replacement patients to the kinesiophobia (TSK ≥ 37) or no-kinesiophobia (TSK < 37) group [16]. The KOOS evaluates five separately scored subscales: pain, symptoms, activities of daily living, recreational and sports-related activities, and quality-of-life [32]. The use of the KOOS has been validated for the assessment of patients after different orthopedic interventions, such as: anterior cruciate ligament reconstruction, meniscectomy and total knee replacement [33]. Higher KOOS scores indicate better outcomes for each subscale. The 12-item OKS is a questionnaire that predominantly focuses on a patient’s ability to perform activities of daily living after knee replacement surgery [34]. Similarly to the KOOS, higher OKS scores indicate better daily functionality.

#### 2.2.3. Performance-Based Measures

The Tinetti test consists of a separate balance and gait assessment score. The balance assessment evaluates the ability of a patient to sit down and rise from a chair, stand with eyes open and closed, and perform more complex coordination tasks that need optimal balance [26]. The gait assessment consists of an evaluation of several variables such as the use of walking aids, gross gait devastations, step length, step width, step symmetry and continuity, gait path, and trunk sways. Each variable can be scored on a 0 to 1 or 0 to 2 scale. A maximum of 12 and 16 points can be scored on the gait and balance assessment, respectively. The sum of the gait and balance assessment scores represents the overall Tinetti outcome. A score lower than 19 out of 28 on the Tinetti indicates poorer physical ability and translates into a five-fold increase of fall risk [29]. A Tinetti score between 19 and 26 also indicates a higher fall risk [35]. A TUG test requires a patient to rise from a chair, walk 3 m towards a mark, return to the chair, and sit down. The outcome is expressed in seconds. The TUG test was used as a PBM since it is highly correlated with a patient’s basic functional mobility. A score of more than 20 s indicates a high risk of falls and poorer functionality [27].

### 2.3. Statistical Analysis

First, Data from both groups (kinesiophobia and no-kinesiophobia) were expressed in terms of mean and standard deviation (SD) of the mean. A Shapiro–Wilk test was performed to test for normality. Since data were normally distributed, an independent Student’s t-test was used to identify significant differences between both groups at discharge and 6 weeks post knee replacement surgery. Statistical significance was set at a α ≤ 0.05. Scatter plots with bivariate regression lines (Pearson’s correlation) were constructed to provide a visual representation of the distribution and correlation between TSK at discharge and PBM outcomes. Finally, multiple linear regression (enter method) was applied to model PBMs outcome (dependent variable) from age, pain (VAS) and TSK-17 outcomes at discharge (independent variables). r^2^-values represent the model’s explanatory power for each dependent variable separately. Standardized beta weights (β) allow a direct comparison of each variable’s contribution to the model. All statistical procedures were performed with the Statistical Package for the Social Sciences (SPSS Inc. version 25, Chicago, IL, USA).

## 3. Results

### 3.1. Independent Sample t-Test on PROMs and PBMs

In this study, 55.8% of knee replacement patients (*n* = 24) suffered from high levels of kinesiophobia at discharge from the hospital and were subsequently allocated to the kinesiophobia group. The independent sample t-test comparison between the kinesiophobia and no-kinesiophobia groups demonstrated a significant difference in PROMs at discharge. Patients with kinesiophobia demonstrated significantly worse pain outcomes (11.84%, *p* = 0.03) on the KOOS pain subscale, compared to patients without kinesiophobia. No other significant differences were observed for PROMs between patients with and without kinesiophobia at discharge or follow-up, except for TSK outcomes at discharge, which served as an allocation variable (Table 2).

For the PBMs, a small but significant difference was observed for the Tinetti test at discharge (mean difference = 1.70, *p* = 0.05). More particularly, a significantly lower Tinetti gait outcome score at discharge was found for the group with a high level of kinesiophobia (mean difference = 1.65, *p* = 0.01). At follow-up, no significant difference for overall Tinetti score was found. Yet, patients with high levels of kinesiophobia still demonstrated a trend towards a lower Tinetti gait outcome (*p* = 0.05), and still have an overall Tinetti outcome that indicates an increased fall risk. No significant differences were found for the Tinetti balance assessment, either at discharge or at follow up. At follow-up, a significant difference could be observed between groups, in which patients with a high level of kinesiophobia needed on average 2.84 s more to execute the TUG test (*p* = 0.03), which is also clinically relevant given the MCD of 2.1 s [36].

### 3.2. Bivariate Analyses and Multiple Linear Regressions

**Figure 1:**
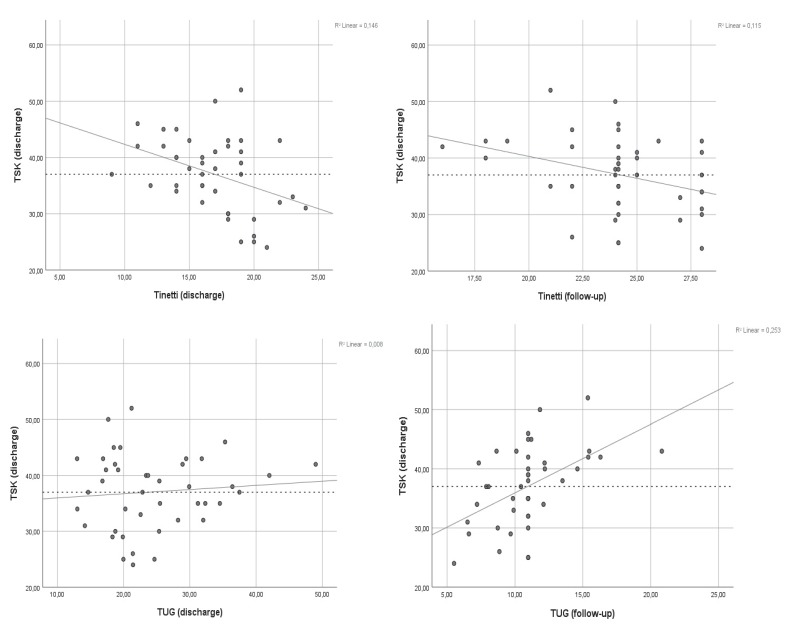
Scatter plots with regression line, showing the relation between TSK scores at time of discharge and PBM outcomes at discharge and follow-up.

Scatter plots of the Tinetti and TUG test at discharge and follow-up are presented in Figure 1.

The multiple linear regression analyses (Table 3) demonstrated low to moderate correlations between kinesiophobia at discharge and the outcomes of the TUG and Tinetti test (range = 0.231–0.464). The explanatory power across models varied between the TUG and Tinetti test, with slightly lower values observed for Tinetti (r² range = 0.215–0.274). A large contribution of TSK outcomes could be observed across regression models for the TUG and Tinetti tests at discharge and follow-up (β range Tinetti = −0.348, −0.349; β range TUG = 0.483, 0.541). Results from the multiple linear regression are presented in Table 3.

## 4. Discussion

The main finding of the present study was the negative influence of kinesiophobia on functional recovery in patients with knee replacement. At time of discharge, patients with a high level kinesiophobia demonstrated lower Tinetti outcomes compared to patients without kinesiophobia. More specifically, this overall difference in Tinetti outcome was due to a significantly worse gait score in patients with kinesiophobia. Furthermore, a significantly worse outcome for the TUG test in knee replacement patients with kinesiophobia could be observed at 6 weeks’ follow-up. This implies that clinicians should be aware of the increased risk of falling among patients with knee replacement at time of discharge.

Findings of this study add body to the current evidence that demonstrates that kinesiophobia, among other psychological factors, can delay or hinder functional recovery in patients who have undergone orthopedic surgery [6,8,9]. Especially since 60% of the increase in pain score could be related to the presence of kinesiophobia [21]. This was also supported by findings from a large cohort study in which it was demonstrated that kinesiophobia negatively influences the mental state, function and the ability to participate in social activities in patients with TKA [21]. This also corroborates with the fear avoidance model of the pain framework, which suggests that patients with a clear tendency towards movement or pain-related fear after an injury are more at risk for depression and disuse [37]. Furthermore, patients suffering from kinesiophobia may, in order to prevent a future injury, develop hypervigilance. This state has been identified as a crucial contributor to the development of chronic pain [38,39]. Also, psychosocial factors are known to influence the biological processes that are crucial for recovery. For instance, neuroplastic changes in central nervous system regions associated with the processing of sensory information, emotions and pain are more likely to occur in patients with kinesiophobia [40,41]. These changes interfere with the normal neurophysiological modulation of pain, and will in turn cause more pain-awareness and disability [13]. Moreover, both psychological and biological alterations contribute to central sensitization and the development of chronic pain [13]. Considering the evidence in which it has already been demonstrated that nearly 25% of patients with a knee replacement suffer from chronic pain, these psychological and biological alterations need to be considered by clinicians when planning treatment. This may lead to better outcomes in patients following knee replacement surgery [42,43]. However, our findings do not indicate that a high level of kinesiophobia negatively affects pain outcomes, since a similar decrease in pain scores could be observed at follow-up between patients with and without kinesiophobia. Yet, given the complexity of pain and pain-management, it is recommended that future studies include other biologic and psychosocial variables such as: inflammation, infection, depression, and catastrophizing [21,44,45]. This will allow a deeper understanding of the pain processes at hand in patients that are recovering from knee replacement.

The recovery of function is a primary goal for patients who have undergone knee arthroplasty surgery [2]. In this study, no significant differences could be found regarding the presence of kinesiophobia and self-reported functionality. However, KOOS ADL and QOL subscales demonstrate clinically relevant, lower outcomes in patients with kinesiophobia. For instance, a difference of 11.8% was observed for the KOOS pain score, which is significantly larger than the minimal clinically relevant difference (MCD) of 6.11% (or 2.2 points). Perhaps, the statistical analysis failed to detect significant differences between groups due to the small sample size. Though, as suggested by previous authors, PROMs might not fully capture the actual functional capabilities of a patient, and should therefore not be interpreted as stand-alone criteria for decision making but rather as an added value to the outcome of PBMs [24,25]. In our patient group, we could observe significantly lower scores at time of discharge for the Tinetti and, more particularly, the Tinetti gait score in patients with high levels of kinesiophobia. This suggests that patients with a TSK outcome ≥ 37 have even a higher fall risk than the average patient with knee replacement, knowing that patients in the no-kinesiophobia group also demonstrated Tinetti scores below the threshold of 19, indicating an increased risk for falling. Even at 6 weeks’ follow-up, both groups’ scores were below the threshold of 26, indicating an elevated level of fall risk. At both measurements, the increased fall risk was likely due to gait abnormalities since the Tinetti gait test was found to be significantly lower, which was not the case for the balance assessment. These findings are in agreement with previous research performed in other knee pathologies, showing that patients with kinesiophobia are more prone to develop gait deviations and subsequently have an increased fall risk [46]. Clinicians should therefore be aware of the relation between kinesiophobia and the risk of falling, especially since fall incidents are a major cause for complications and readmission in patients with a knee replacement [20]. However, in order to further support this statement, more studies are necessary in which the causal relation between (fall-related) readmission risk and kinesiophobia are addressed.

The time needed to perform the TUG test is a highly indicative measure of function, as it requires a patient to perform several postural and dynamic transitions needed during activities of daily living (e.g., sit to stance, stance to sit and initiating and stopping gait) [27,29]. In this study, we observed that patients with a high level of kinesiophobia needed more time to complete the TUG test at discharge than those without kinesiophobia. The difference of 1.45 s, even though not statistically significant, is clinically relevant. Furthermore, both groups scored above the 20 s threshold, indicating reduced functionality and a higher fall risk in both groups at time of discharge. At the 6 weeks’ follow-up, patients in the kinesiophobia group needed significantly more time to complete the TUG test. However, both groups demonstrated TUG outcomes below the required threshold at that time. These findings are in accordance with previous studies in which a relation was demonstrated between kinesiophobia and the willingness or ability to perform transitions during activities of daily living [47,48]. Based on the proposed findings, one can conclude that a high level of kinesiophobia at time of discharge may negatively affect the short-term performance of the TUG test in patients with knee replacement.

Some limitations should be considered when interpreting the results of the present study. The relatively small sample size makes it hard to generalize results. However, the rule of thumb concerning the regression analysis, in which it is stated that at least 10 subjects are included per factor, was respected. Nonetheless, larger sample size studies are warranted. Also, a single surgeon performed all knee replacements using the same surgical procedure and according to the clinical path specific to the hospital. Nevertheless, this allowed for the control of several confounding factors associated with multi-center studies and resulted in a unique dataset. It should be noted that the proposed cohort included patients with UKA and TKA, and that it has been suggested that UKA patients more easily recover function than those with TKA. Yet in the current study, an additional analysis demonstrated no significant differences for PROMs and PBMs between patients with UKA and TKA. Perhaps, future studies should focus on the impact of kinesiophobia on the recovery of function following UKA and TKA. Another factor that needs to be taken into consideration when interpreting the results of the current study is aging. It is well-known that ageing is associated with a higher risk of falling and a reduced functionality [49]. Bearing in mind that the average age of the included patients was 65 years, this could have influenced the outcome of the proposed results. Nevertheless, the included patients with kinesiophobia demonstrated significantly worse outcomes at discharge and follow-up compared to the average TKA population [20], which suggests that kinesiophobia did contribute to the increase in fall risk and reduced functionality.

The findings reported in this study are of great clinical value, considering the high prevalence (55.8%) and the impact of kinesiophobia on the short-term recovery of patients who have undergone knee replacement surgery. Based on this study, the negative influence of kinesiophobia on the biopsychosocial outcome and physical performance of knee replacement patients at the early stage of rehabilitation should receive further attention. Hence, the identification of patients with elevated levels of kinesiophobia will allow clinicians to set out a personalized treatment plan, in accordance to the specific needs of the patient, which should result in better outcomes following knee replacement surgery [50]. For instance, cognitive-behavioral strategies are reported to be highly successful treatment options for the reduction of symptoms (e.g., pain and disability) induced by the psychological state of a patient [51]. Another possible intervention was demonstrated in a recent randomized clinical trial, where wearing a knee brace for two weeks resulted in a 5-point reduction in TSK scores in women with patellofemoral pain [52]. This cost-effective and easily implemented intervention could potentially result in similar outcomes in knee replacement patients and might therefore be considered in future research. The higher risk of falling that was observed among knee replacement patients with a high level kinesiophobia, should also be taken into account by clinicians, especially since fall incidents are the primary reason for hospital readmission after knee replacement surgery [20].

Considering the clinical relevance of the proposed findings, we suggest that more research on the impact of kinesiophobia and other psychosocial factors on the recovery process needs to be conducted. Especially in the knee arthroplasty population, since these patients will benefit from an individualized and multidisciplinary treatment approach [53]. More specifically, anxiety and kinesiophobia seem to be crucial roles in the rehabilitation and recovery process [54]. It is therefore our strongest belief that more studies investigating the influence of psycho-cognition on recovery after knee replacement surgery are warranted.

## 5. Conclusions

This study indicates that patients who have undergone knee replacement surgery may present high levels of kinesiophobia at time of discharge from the hospital. It appears that this psycho-cognitive factor could play a role in the recovery process of these patients, as it may negatively influence short-term performance-based outcomes.

## Figures and Tables

**Table 1:** Subjects’ characteristics.

Characteristics	Mean ± SD
Sex	M = 24, F = 19
Age (years)	65.17 ± 7.33
Weight (kg)	82.25 ± 13.53
BMI (kg/m²)	27.61 ± 4.79
Arthroplasty type	UKA = 14, TKA = 29

M= male; F= female; SD= standard deviation; BMI= body mass index; kg= kilogram; m= meter; UKA = unicondylar knee arthroplasty; TKA = total knee arthroplasty.

**Table 2:** Patient-reported and performance-based outcome at 3 days and 6 weeks post knee replacement surgery.

PROMs KOOS	Discharge (3 days)			*p*-Value	Follow-Up (6 weeks)			*p*-Value
	Kinesiophobia (*n* = 24)	No-kinesiophobia (*n* = 19)	Mean Difference (95% CI)		Kinesiophobia	No-kinesiophobia	Mean Difference (95% CI)	
Pain (%)	45.89 ± 6.31	57.73 ± 5.51	11.84 (−8.00, −5.15)	0.03	66.20 ± 8.81	75.33 ± 5.73	9.13 (−9.82, 3.06)	0.29
Symptoms (%)	57.13 ± 5.41	60.11 ± 3.66	2.98 (−2.63, 2.19)	0.85	67.00 ± 2.84	70.72 ± 3.58	3.72 (−4.53, 1.39)	0.41
ADL (%)	52.95 ± 11.63	61.77 ± 9.99	8.82 (−13.61, 0.48)	0.06	78.12 ± 10.67	81.62 ± 9.41	3.49 (−10.58, 4.94)	0.57
QOL (%)	40.25 ± 2.78	51.13 ± 3.22	1.74 (−2.99, 2.67)	0.07	65.13 ± 3.44	66.25 ± 3.32	1.12 (−3.70, 0.22)	0.89
OKS (0–48)	26.04 ± 8.65	30.06 ± 7.07	4.01 (−1.04, 9.06)	0.12	36.06 ± 7.61	38.00 ± 6.55	1.94 (−4.07, 7.96)	0.51
TSK (17–60)	41.83 ± 3.95	30.89 ± 3.53	10.95 (−13.35, −8.69)	0.01	36.37 ± 6.78	32.90 ± 6.44	3.47 (−9.01, 2.06)	0.21
VAS (0–10)	4.46 ± 1.88	3.94 ± 1.51	0.51 (−1.61, 0.59)	0.35	1.89 ± 1.45	2.00 ± 1.09	0.11 (−0.93, 1.16)	0.83
PBMs								
Tinetti (0–28)	16.08 ± 2.09	17.78 ± 2.77	1.69 (−0.26, 3.65)	0.05	24.00 ± 6.89	25.75 ± 4.41	1.75 (−0.10, 5.32)	0.78
Gait (0–12)	6.30 ± 2.20	7.95 ± 2.10	1.56 (0.21, 2.93)	0.01	9.40 ± 2.19	10.1 ± 2.56	0.66 (−1.28, −2.61)	0.05
Balance (0–16)	9.70 ± 1.88	9.94 ± 1.95	0.23 (−0.97, 1.44)	0.69	14.60 ± 2.21	14.03 ± 2.20	0.56 (−0.77, 2.88)	0.82
TUG (s)	25.04 ± 9.39	23.59 ± 6.57	1.45 (−5.43, 0.27)	0.58	12.18 ± 3.59	9.34 ± 2.03	2.84 (−5.92, −1.50)	0.03

PROMs = patient-reported outcome measures; PBMs = performance-based measures; SD = standard deviation; KOOS = knee osteoarthritis outcome score (%); TSK = Tampa scale for kinesiophobia (min. 17, max. 68); OKS = oxford knee score (min. 12, max. 60); ADL= Activity of Daily Living; QOL = Quality Of Life; VAS = visual analogue scale (min. 0, max. 10; PBMs= performance-based measures; TUG = Timed Up and Go, CI= confidence interval. **Bold** indicates significant differences

**Table 3:** Multiple linear regression analyses.

	r	r²	Standardized β	*p*-Value
TUG (discharge)				
Model	0.234	0.530	-	0.54
TSK			0.483	0.004
VAS pain			0.157	0.341
Age			0.113	0.494
TUG (follow-up)				
Model	0.653	0.427	-	0.05
TSK			0.541	0.004
VAS pain			0.202	0.241
Age			0.122	0.439
Tinetti (discharge)				
Model	0.464	0.215	-	0.01
TSK			−0.348	0.025
VAS pain			−0.242	0.175
Age			−0.145	0.332
Tinetti (follow-up)				
Model	0.490	0.274	-	0.04
TSK			−0.349	0.066
VAS pain			−0.242	0.193
Age			−0.178	0.323

Multiple linear regression analysis (enter method) was used to model performance-based outcomes from age, pain at time of the test, and Tampa scale for kinesiophobia scores at time of discharge. r indicates the correlation (Pearson’s) between the model and dependent variable; r² represents the model explanatory power with according standardized β weights, showing the contribution of each independent variable to the proposed model; TUG = Timed Up and Go test; VAS pain =Visual Analogue Scale for pain; TSK = Tampa Scale for kinesiophobia outcome at time of discharge from the hospital.
